# Unexpected Detection of Abscessualized Lung Carcinoma on Tc-99m-HMPAO-labeled Leukocytes Scintigraphy Misdiagnosed on Chest Computed Tomography

**DOI:** 10.4274/mirt.galenos.2020.54366

**Published:** 2021-02-09

**Authors:** Laura Cosma, Viviana Frantellizzi, Mariano Pontico, Giuseppe De Vincentis

**Affiliations:** 1 Sapienza University of Rome, Department of Radiological Sciences, Division of Oncology and Anatomical Pathology, Rome, Italy; 2 Sapienza University of Rome, Department of Molecular Medicine, Rome, Italy; 3 Sapienza University of Rome, Ph.D. Program in Morphogenesis and Tissue Engineering, Department of Medico-Surgical Sciences and Biotechnologies, Rome, Italy

**Keywords:** Tc-99m-HMPAO-labeled leukocytes, hybrid imaging, SPECT/CT, FUO, abscess, abscessualized cancer

## Abstract

Technetium-99m (Tc-99m)-hexamethylpropylene amine oxime (HMPAO)-labeled leukocytes scintigraphy is well established for investigating and diagnosing infections in bone and soft tissue, as well as for the detection of occult infection. A 71-year-old female who was recently diagnosed with bronchopulmonary neuroendocrine tumor of the right lung was referred for an intermittent fever of unknown origin associated with chill at night for the last month. Chest computed tomography (CT) scan showed a thrombotic widespread of the superior vena cava and a solid pathological tissue in the superior segment of the inferior lobe of the right lung with consensual atelectasis. Being a carrier of port-a-cath, an infection of this device was suspected. Therefore, Tc-99m-HMPAO-labeled leukocytes single-photon emission computed tomography (SPECT) was performed, and matching pairs of CT scan and Tc-99m-HMPAO-labeled white blood cell SPECT images were fused. Through this means, it was found that the area of the radiotracer increased uptake corresponded with the soft tissue density mass detected by CT scan localized at the inferior lobe of the right lung. The hybrid SPECT/CT fused imaging was crucial for diagnosis of the presence of a lung abscess localized in correspondence with the known lung cancer region.

## Abstract (tr)

Teknesyum-99m (Tc-99m)-heksametilpropilen amin oksim (HMPAO) işaretli lökosit sintigrafisi, kemik ve yumuşak dokudaki enfeksiyonları araştırmak ve teşhis etmek ve ayrıca gizli enfeksiyonu saptamak için iyi bir şekilde tasarlanmıştır. Yakın zamanda sağ akciğerde bronkopulmoner nöroendokrin tümörü teşhisi konan 71 yaşındaki bir kadın, son bir ay içinde geceleri üşüme ile ilişkili bilinmeyen kaynaklı aralıklı ateş nedeniyle sevk edildi. Toraks bilgisayarlı tomografisi (BT) taraması, üst vena kavanın trombotik yayılımını ve sağ akciğerin alt lobunun üst segmentinde karşılıklı atelektazisi olan katı patolojik dokuyu gösterdi. Bir kateter portu taşıyıcısı olduğundan, bu cihazın bir enfeksiyonundan şüpheleniliyordu. Bu nedenle, Tc-99m-HMPAO işaretli lökosit tek foton emisyonlu bilgisayarlı tomografi (SPECT) gerçekleştirildi ve eşleşen BT taraması ve Tc-99m-HMPAO işaretli beyaz kan hücresi SPECT görüntü çiftleri birleştirildi. Bu yolla, artmış radyofarmasötik tutulumun, sağ akciğerin alt lobunda lokalize BT taraması ile tespit edilen yumuşak doku yoğunluğu kütlesine karşılık geldiği bulundu. Hibrid SPECT/BT füzyon görüntüleme, bilinen akciğer kanseri bölgesi ile uyumlu olarak lokalize edilmiş bir akciğer apsesinin varlığının teşhisi için çok önemliydi.

## Figures and Tables

**Figure 1:**
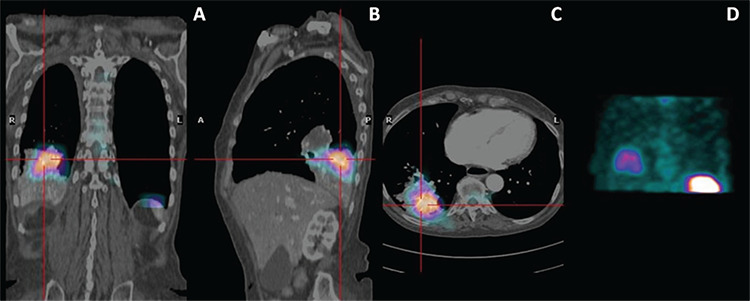
Fused technetium-99m (Tc-99m)-hexamethylpropylene amine oxime (HMPAO)-labeled leukocytes single-photon emission computed tomography/CT (SPECT/CT) hybrid images on coronal (A), sagittal (B), and transaxial (C) planes with Tc-99m-HMPAO-labeled leukocytes chest SPECT coronal maximum intensity projection (D). Principal clinical indications for Tc-99m-HMPAO-labeled leukocytes scintigraphy include inflammatory bowel disease, osteomyelitis, soft tissue sepsis, and occult fever (1,2). Autologous leukocytes are characterized by high specificity because they only accumulate into the inflamed tissues due to active migration and because very infrequent fixation occurs in neoplastic tissues (1,3). A 71-year-old Caucasian female was admitted to our department for an intermittent fever of unknown origin associated with chill at night for the last month. She was recently diagnosed with bronchopulmonary neuroendocrine tumor at the right lung superior segment of the inferior lobe. A phlebitic process of intravenous catheter was also depicted. Chest CT scan showed a thrombotic widespread of the superior vena cava (SVC) and another one near the infusion catheter localized in the proximal tract of the brachiocefalic artery. Moreover, in the superior segment of the inferior lobe of the right lung, a conglobated solid pathological tissue (50x41 mm^2^) causing thickening and infiltration of the scissural pleura and incorporating the segmental bronchial vessels, particularly the bronchial branches of the postero-basal segment, almost completely obliterated with consensual atelectasis of the segment was reported. Being a carrier of a port-a-cath with the catheter guided into the SVC, an infection of this device or the thrombus described above was suspected. Transthoracic and transesophageal echocardiogram were negative for the infectious source on the SVC catheter and tested blood cultures of all microorganisms. Tc-99m-HMPAO-labeled leukocytes scintigraphy was performed in order to identify the source of infection responsible for her clinical course. Whole body planar and SPECT images of the chest region were acquired. Matching pairs of CT scan and Tc-99m-HMPAO-labeled white blood cell SPECT images were fused using dedicated Xeleris software (GE Healthcare) to generate hybrid images of overlying transmission and emission data. The three-plane triangulation showed the clear match between the densitometric alteration found on CT scan and the markedly pathologic Tc-99m-HMPAO-labeled leukocytes uptake in the inferior lobe of the right lung, thus unveiling the presence of an active infectious disease.

**Figure 2:**
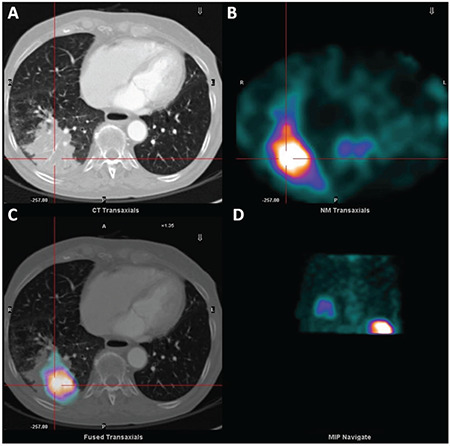
CT scan (A), Tc-99m-HMPAO-labeled leukocytes chest SPECT (B), fused Tc-99m-HMPAO-labeled leukocytes SPECT/CT hybrid image (C), and chest SPECT maximum intensity projection (D) on transaxial planes. SPECT images showed an area of clearly increased tracer uptake on the right inferior region of the chest, which was more evident in the delayed 24 h images. Fused Tc-99m-HMPAO-labeled leukocytes SPECT and CT scan of the chest demonstrated that the area of increased uptake corresponded with the soft tissue density mass (detected by CT scan) localized at the inferior lobe of the lung (Figure 1, 2). The hybrid SPECT/CT fused images were crucial in order to achieve the right interpretation of this case (4), thus enabling the taken of an informed decision regarding the diagnosis of the presence of a lung abscess localized exactly in correspondence with the known lung cancer region. Therefore, the presence of SCV catheter infection was safely excluded. A proper match between the CT densitometric alteration and the pathologic Tc-99m-HMPAO-labeled leukocytes uptake in the inferior lobe of the right lung was clearly evident. Based on the findings obtained, antibiotic therapy was administered (amoxicilline and clarithromicine for ten days), and the patient’s clinical conditions improved during treatment. Also, a concomitant Erythrocyte Sedimentation Rate and Polymerase Chain Reaction values decline was observed, followed by complete normalization after two weeks from onset of the treatment.
